# Experiences of a Web-Based Quality of Life Self-Monitoring Tool for Individuals With Bipolar Disorder: A Qualitative Exploration

**DOI:** 10.2196/16121

**Published:** 2019-12-04

**Authors:** Emma Morton, Rachelle Hole, Greg Murray, Simone Buzwell, Erin Michalak

**Affiliations:** 1 Department of Psychiatry University of British Columbia Vancouver, BC Canada; 2 School of Social Work University of British Columbia Okanagan, BC Canada; 3 Department of Psychological Sciences Swinburne University of Technology Hawthorn Australia

**Keywords:** bipolar disorder, self-monitoring, self-management, qualitative, recovery, quality of life, eHealth

## Abstract

**Background:**

Self-monitoring of symptoms is a cornerstone of psychological interventions in bipolar disorder (BD), but individuals with lived experience also value tracking holistic outcomes, such as quality of life (QoL). Importantly, self-monitoring is not always experienced positively by people with BD and may have lower than expected rates of engagement. Therefore, before progressing into QoL tracking tools, it is important to explore user perspectives to identify possible risks and benefits, optimal methods to support engagement, and possible avenues to integrate QoL self-monitoring practices into clinical work.

**Objective:**

This study aimed to conduct a qualitative exploration of how individuals with BD engaged with a Web-based version of a BD-specific QoL self-monitoring instrument, the QoL tool.

**Methods:**

A total of 43 individuals with BD engaged with a self-management intervention with an optional Web-based QoL self-assessment tool as part of an overarching mixed method study. Individuals were later interviewed about personal experiences of engagement with the intervention, including experiences of gauging their own QoL. A thematic analysis was used to identify salient aspects of the experience of QoL self-monitoring in BD.

**Results:**

In total, 4 categories describing people’s experiences of QoL self-monitoring were identified: (1) breadth of QoL monitoring, (2) highlighting the positive, (3) connecting self-monitoring to action, and (4) self-directed patterns of use.

**Conclusions:**

The findings of this research generate novel insights into ways in which individuals with BD experience the Web-based QoL self-assessment tool. The value of tracking the breadth of domains was an overarching aspect, facilitating the identification of both areas of strength and life domains in need of intervention. Importantly, monitoring QoL appeared to have an inherently therapeutic quality, through validating flourishing areas and reinforcing self-management efforts. This contrasts the evidence suggesting that symptom tracking may be distressing because of its focus on negative experiences and positions QoL as a valuable adjunctive target of observation in BD. Flexibility and personalization of use of the QoL tool were key to engagement, informing considerations for health care providers wishing to support self-monitoring and future research into Web- or mobile phone–based apps.

## Introduction

### Background

Bipolar disorder (BD) is a chronic mood disorder characterized by periods of mania (irritable or elevated mood with increased activity) and depression, experienced by up to 2.4% of the global population [1]. Self-monitoring of symptoms is an active ingredient of psychosocial therapies for BD [2], and it is recommended as an adjunctive strategy in treatment guidelines [3]. People with lived experience of BD have expressed interest in tracking additional life domains. Quality of life (QoL), a holistic outcome, has strong potential to meet this market need. Before progressing into QoL tracking apps, it is important to attend to the evidence suggesting that self-monitoring in BD is not always experienced positively. Attention to symptoms may be a distressing reminder of being unwell [4] and may reinforce depressive symptoms [5]. Given its broad focus and attention to both positive and negative experiences, QoL may be an acceptable target for self-monitoring and play a valuable complementary role to symptom tracking. This qualitative study is an initial step toward understanding self-monitoring of QoL in people with BD by exploring user experiences of a Web-based BD-specific QoL measurement instrument.

Self-monitoring of symptoms is a cornerstone of psychological therapies for BD used to support self-management, detect early warning signs of relapse, and guide appropriate intervention. Traditionally, such interventions have focused on sleep and mood, which have been shown to improve time to recurrence of mood episodes, rates of hospitalization, and functioning [6]. However, there is evidence to suggest that people with BD are interested in tracking other life domains. A survey of self-monitoring strategies used by people with BD indicated that although mood and sleep were most commonly monitored, individuals also tracked a variety of other areas including finances, social interactions, substance use, household management, pet care, and leisure time [7]. Individuals created *elaborate* methods to track multiple indicators, suggesting a market need for apps facilitating simultaneous tracking of diverse life domains. QoL is a broad construct taken to represent aspects of functioning and satisfaction in occupational, environmental, social, physical, and psychological aspects of life [8]. QoL may, therefore, represent a prime outcome that addresses the desire for more inclusive forms of self-monitoring in BD. Furthermore, individuals with BD have nominated QoL as an outcome that is prioritized alongside or even *above* symptom reduction [9-14]. Increasing focus has been placed on QoL-focused self-management strategies and psychosocial interventions for BD [15,16], of which, QoL tracking would form a natural complement. Although studies on the validity, feasibility, and acceptability of routine QoL self-assessment have been conducted in oncology [17-19], this experience remains poorly understood in mental health.

Despite research indicating positive effects of self-monitoring in BD, those with lived experience may be ambivalent or inconsistent about daily assessment. Rates of adherence to self-monitoring interventions in BD vary widely, with missing data rates between 6% and 58% for electronic methods [20,21]. Although some disengagement is expected given the effort required [22], this may not fully explain adherence rates. Studies in BD and other psychiatric conditions suggest that there are additional psychological costs to self-monitoring when living with a chronic mental illness. One such qualitative study specifically explored experiences of using the Life Chart Methodology (LCM), a daily record of manic and depressed mood, sleep duration, number of mood changes, and medication use [23], in individuals with bipolar I disorder (BD-I) [4]. The usage of the LCM was perceived by almost half (43%) of the participants as an unpleasant reminder that they were living with BD, forcing confrontation with the limitations of illness. Some believed that focusing on negative experiences increased their depression, and this was associated with a reluctance to use the LCM. The authors theorized that aversive experiences of the LCM were linked the psychological threat of losing the identity of the *healthy self*. Although quantitative research has not explored these experiences on a larger scale, there is some support for the idea that self-monitoring may reinforce negative experiences. Indeed*,* a study of daily BD mood tracking using mobile phone technology was shown to increase depressive symptoms in the intervention group [5]. In addition, in digital health interventions for psychosis, qualitative studies and adverse event reports suggest that routine self-assessment of symptoms may increase rumination on negative experiences, generate fear of relapse when negative changes in mental health are observed, or trigger distressing memories of episodes of psychosis [24,25].

### Objective

It is not known whether QoL self-monitoring would be associated with a similar impact as symptom tracking in BD. Qualitative exploration of such applications may answer questions that quantitative studies of feasibility and acceptability may not, including possible risks and benefits, optimal integration of QoL data into clinical practice, and strategies to support engagement with self-assessment tools [25]. The aim of this study was to conduct a qualitative exploration into how individuals with BD engaged with a Web-based version of a BD-specific QoL self-monitoring instrument, the QoL tool [26].

## Methods

### Design

**Figure 1:**
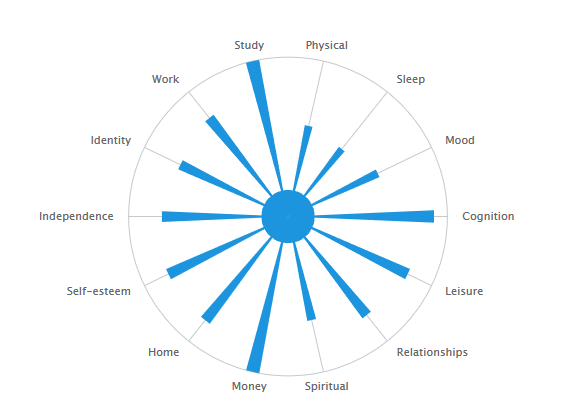
Representation of quality of life tool results.

By using descriptive qualitative methods [27], this study was conducted within the context of a broader mixed method (ie, both quantitative and qualitative) project investigating the impact of self-management in BD [16]. The aim of the overarching project was to investigate the effectiveness of a website (Bipolar Wellness Centre [28]) and various associated information delivery modalities for communicating and encouraging the use of various self-management strategies (for a detailed description of these, see Michalak et al. [16]). The Bipolar Wellness Centre linked to the externally hosted QoL tool [29], which contains a Web-based version of a BD-specific QoL self-report measure developed through a Community-Based Participatory Research process. Life domains important to the experience of QoL in BD were elicited through interviews with individuals with BD, their family members, and health care providers [30]. The final instrument assesses satisfaction in the last week across domains of relationships, home duties, leisure activities, physical health, sleep, mood, money, independence, spirituality, self-esteem, cognition, identity, and work or study, if relevant [26]. Questionnaire items are positively framed, that is, respondents are asked about their level of enjoyment and positive experiences across domains, rather than distress or problems. Results from the QoL tool are represented graphically, and the quantitative summary of scores gives the user an optional link to the corresponding page of the Bipolar Wellness Centre (Figure 1). An optional introductory video describes these features and the developer’s recommendations for use (completing the measure weekly). Participants in the primary mixed method study were free to choose whether they would complete the QoL tool. To address the aim of this study to explore experiences of self-monitoring QoL, the focus was on the participants’ description of engagement with the QoL tool rather than specific self-management strategies or aspects of the Bipolar Wellness Centre that they may have also used.

### Recruitment

The participants for this study were a subsample of those in an overarching project who agreed to participate in a follow-up qualitative telephone interview. Participants in the primary project were required to meet the following criteria: (1) aged 19 years or above, (2) able to communicate in English, (3) able to provide informed consent, (4) a resident of Canada, and (5) have a self-reported diagnosis of BD. Self-reported diagnoses of BD was considered sufficient, given that this is typically congruent with the results of formal diagnostic interviews [31].

Recruitment in the overarching project was conducted via the following: (1) notices were sent to participants in prior BD studies in the Mood Disorders Centre, Department of Psychiatry (University of British Columbia), who consented to be contacted in the future regarding new studies of potential interest, (2) health care providers affiliated with the Collaborative Research Team for Psychosocial Issues in BDs (CREST.BD) network were provided with information about this project and asked to place informational leaflets in their waiting room, and (3) advertisements in print (eg, community newspapers) and Web-based (eg, blogs) media.

At enrollment in the project, individuals indicated their consent to be contacted at a later date regarding participation in a telephone interview. For the qualitative arm, purposive sampling was used to identify consenting participants, who were then contacted via email and invited to participate in the qualitative interview. Participants received a Can $20 honorarium in the form of a gift certificate as compensation for time spent in the interview.

Purposeful criterion sampling [32] was used to establish a sample that reflected the diversity of the primary project sample, namely, demographics and engagement with the various components of the Bipolar Wellness Centre. Participants were specifically recruited to ensure representation of various age groups, gender, BD subtype, and experiences with the spectrum of self-management strategies across the information delivery modalities. For the purposes of this study, these sampling criteria were not applied in the analytic framework or the description of the findings, as focus was on experiences of engaging with the QoL tool.

### Qualitative Interview

A semistructured interview schedule was developed based on 3 sets of topics informed by the overarching project aim (engagement with information on self-management strategies, implementation of self-management strategies, and perceived impact on QoL). Main findings have been published elsewhere [16,33,34]. Germane to this subanalysis, participants were asked if they had used the QoL tool or had any plans to use it in future. They were also asked whether using the QoL tool had any impact on their engagement with the Bipolar Wellness Centre (eg, “Did completing the QoL tool have any impact on the kinds of changes you thought about making in your life?”) or their perceived QoL (eg, “Did completing the QoL tool impact your QoL in any way?”). Probes and reflective listening were used to elicit depth in participant responses.

### Procedure

The Behavior Research Ethics Board of the University of British Columbia approved the study. All participants received written information on the study and gave written consent to be contacted for an interview. Data in the study were treated confidentially, and transcripts were deidentified.

Consenting participants were contacted via email approximately 2 weeks after participating in the self-management intervention to schedule a telephone interview. In total, 67 participants were invited to participate in the qualitative interview; of these, 24 declined to participate or did not respond. A total of 43 interviews were conducted by the first author. Interviews lasted from 20 to 70 min (mean 39.4 min, SD 11.2). They were digitally recorded and transcribed verbatim by the first author (24/43, 55%) or research assistants (checked by the first author for accuracy; 19/43, 44%).

### Data Analysis

Braun and Clark’s [35] guidelines for thematic analysis were followed. A thematic analysis provides a flexible research tool that can provide a rich, detailed, and complex account of patterns across an entire dataset. As the primary aim of the interview schedule was to explore experiences of the self-management intervention and perceptions of QoL, there was relatively limited discussion of participants’ experiences of engaging with the QoL tool. Consequently, findings are presented at the level of *descriptive, literal categories* rather than interpretative and abstracted themes [35].

The first author was familiarized with the data through the process of transcribing interviews and re-reading transcripts. Data were assigned brief descriptive codes (meaning units) in the qualitative data management software NVivo (QSR International, 2016). Codes were examined, and descriptive categories were generated [35]. The content of categories was reviewed for coherency, and transcripts were revisited as categories were developed to ensure that all relevant data were adequately captured and described. The essence of the most salient categories in relation to this aim has been summarized with illustrative transcript extracts. To reduce the risk of bias and address analytic validity, coauthor EM reviewed both the descriptive accounts of categories and transcripts for coherency, with disagreements resolved via consensus.

## Results

### Participants

A total of 43 participants were interviewed for the qualitative study (42.2% of the primary study sample). The modal age range was 45 to 54 years. Most participants (30/43, 69%) were female. The most frequently reported diagnosis was BD-I (24/43, 55%), followed by bipolar II disorder (BD-II) (16/43, 37%). A participant reported a diagnosis of rapid cycling BD-II, and 2 participants reported a diagnosis of bipolar disorder not otherwise specified (BD-NOS).

### Overview of Key Findings

Of the interviewed sample, 74% (n=32/43) reported using the QoL tool. In total, 4 key categories are described: (1) breadth of QoL monitoring, (2) highlighting the positive, (3) connecting self-monitoring to action, and (4) self-directed patterns of use.

### Breadth of Quality of Life Monitoring

A predominant experience of QoL self-monitoring was the value of reporting satisfaction across a range of life domains. The QoL tool was described as supporting users to attend to a greater range of life domains than they may otherwise monitor, by reminding people of important areas and increasing the granularity with which they appraised their circumstances:

It has helped me look at the different domains and pay attention... it’s all one big thing, but now that it’s in domain names it’s kind of easier to organize and pay attention to those domains.

Male, aged 35-44 years, BD-I

Receiving a breakdown of scores across domains was described as helping people consider the influence of each aspect of QoL separately, which could facilitate the identification of areas in need of improvement that are otherwise masked by an overall feeling of well-being:

It’s really good having that separation into categories in terms of thinking of each one independently of the other... even when you feel like overall you’re doing well, there could be one thing that, when you actually sit down and honestly think about it, you may discover that’s an area you may need to work on more.

Male, aged 45-54 years, BD-II

For some participants, the ability to parse the influence of various aspects of QoL supported them in identifying the causes of mood changes. A participant described using QoL tool data to facilitate discussions with their psychiatrist about possible relapse prodromes:

If something is starting to get wonky... it’s easier when you have to talk to your psychiatrist because you can bring it in and say “this is what’s going on, this area is getting affected, these areas are getting affected.”

Female, aged 35-44 years, BD-I

In some cases, the range of domains covered by the QoL tool was explicitly contrasted to traditional forms of self-monitoring in BD. A participant noted that it was difficult to provide a single rating on a mood diary that captured the variety of their day-to-day experiences:

I always hated the mood diaries, it seems too constricting to just rate my mood on a scale of 1 to 10 for one day.

Female, aged 25-34 years, BD-I

The breakdown of results of the QoL tool also reduced the burden of interpreting changes:

The daily mood chart, you’ve got to interpret it, you’ve got to sit there and look back over it... and this tool it’s right there.

Male, aged 55-64 years, BD-I

The breadth of domains was not universally appreciated; in an instance, monitoring traditional outcomes was preferred. A participant described intentions to track their sole area of concern (sleep) instead of using the QoL tool.

### Highlighting the Positive

A number of participants reported that the QoL tool served an important function in highlighting life domains where they were flourishing. Individuals described the QoL tool results as drawing their attention to areas of strength, which was often accompanied by a sense of appreciation or a positive affirmation that one was on the right track:

I feel like the tool is good even when you’re doing well because, any kind of positive, anything that makes me feel good about myself, is always positive... That’s kind of what it feels like, that encouragement, that—yes, you’re doing good, you’re doing a good job.

Male, aged 45-54 years, BD-II

Related to the importance of monitoring a breadth of QoL domains, individuals appreciated being able to note strengths at the same time as identifying areas in need of additional support:

Here’s where I’m satisfied, here’s where I’m not satisfied... there are some things I want to change, but I wasn’t mad at myself when I looked at the results.

Female, aged 25-34 years, BD-II

Tracking strengths could also play a powerful role in identity. An individual described a sense of reclaiming who they were beyond the *sick role* of bipolar by noticing the range of areas where they were performing well:

It made me feel really good actually, because I knew I was on the right track... yes, I suffer from bipolar, and now it’s [participant’s name] and I’m going to work, I’m functioning, full time hours, and able to handle a lot more than I used to.

Female, aged 35-44 years, BD-I

In some cases, reviewing results prompted a positive reappraisal of circumstances. For example, a participant described reflecting on their relationships after they received a higher domain score than predicted:

I saw that and thought... maybe it’s not such a horrible situation, and I think it’s better than I might have thought.

Female, aged 45-54 years, BD-I

Noting positive results was also described as drawing attention to and encouraging continued application of self-management strategies that were supporting the areas of good QoL. This is exemplified by a participant who added additional physical activities to their schedule after reflecting on the positive impact of their existing routine:

It made me appreciate what I was already doing and I kept doing it... but then it made me think about how that is helping me, so I started swimming as well.

Female, aged 25-34 years, BD-I

The connection between QoL tracking and self-management practices is explored in detail in the following section.

### Connecting Self-Monitoring to Action

Over half of those who used the QoL tool described it as guiding their self-management efforts (n=17). Individuals found QoL self-monitoring enabled them to identify areas where they scored lower, which prompted them to either implement self-management strategies or focus their efforts researching ways to improve that specific domain:

Doing the Quality of Life tool is what made me think about my social activities being very weak... based on that awareness I chose to take action on it.

Female, aged 45-54 years, BD-II

Aspects of the display of QoL tool results were flagged as important in connecting self-monitoring to action. For some individuals, the visual aspect of the QoL tool *results* display was particularly important in identifying areas in need of attention. In such cases, the idea of an *unbalanced* wheel was described, as in the following account:

It also made me realize how unbalanced I could be... I was okay in many aspects, and going back to relationships for me, was really bad on that aspect. I kind of knew it but when I saw it represented that way with the spokes, I felt I had to work on this.

Male, aged 45-54 years, BD-I

A participant described the numerical breakdown of scores as an objective indicator motivating them to take action:

When I would get a score of 11 out of 20 on something, it made me read more, investigate further on that particular area because it pointed out to me in a very quantitative way, that it’s not just that you sort of think there’s a problem or something you want to improve on, that it actually shows you with numbers, and that’s pretty concrete.

Female, aged 65-74 years, BD-II

Another aspect specific of the QoL tool (vs the paper-and-pencil version of the instrument) resides in its links to the Bipolar Wellness Centre. A small number of people explicitly described using the links in their results summary (see Figure 1) to access information about relevant self-management strategies for domains of interest:

Afterwards, there’s an article on the website itself that talks about the areas and links to all these other resources... that really helped.

Female, aged 25-34 years, BD-I

For some participants, using the QoL tool on an ongoing basis itself became a self-management strategy because of its ability to remind them to take action:

I do need to be reminded of things every so often, so if I took that quiz more often it would probably help me remind myself.

Female, aged 25-34 years, BD-I

Ongoing plans for QoL self-monitoring are discussed in more detail in the following section.

### Self-Directed Patterns of Use

When asked about their use of the QoL tool (in the weeks since the start of the intervention and plans for future use), most people talked about tracking QoL in an ongoing sense, but the intended patterns of use varied. Some participants described plans for routine use on a weekly, biweekly, or monthly basis. In such cases, QoL self-monitoring was sometimes described as a preventative strategy, for example “to track things before they get so serious” (female, aged 45-54 years, BD-I). Others spoke about the importance of obtaining enough data to provide insight into patterns:

I think it’s important to do the Quality of Life tool on a fairly regular basis, because that’s the only way you can identify changes or trends, not just when you’re at a peak or valley.

Female, aged 45-54 years, BD-II

For others, regular use was important to keep self-management efforts on track:

I try to do it weekly, and it’s a reminder that I have to look after all aspects.

Male, aged 55-64 years, BD-I

Others planned on using the QoL tool infrequently, such as every few months. This was sometimes described as *checking in* on an ad hoc basis to see if progress could be detected:

I certainly will do it again, maybe in a few months again to see if anything has changed, has improved.

Male, aged 35-44 years, BD-II

For others, QoL tracking could be deployed in response to mood changes to gather data about the cause or identify helpful strategies:

There’s a tool that acts as a checklist for me: when I don’t feel great, I can go over that and it helps me.

Male, aged 45-54 years, BD-I

Finally, QoL self-monitoring may only occur if usual forms of gauging wellness were not available (eg, the opinion of a trusted health care provider when going on holiday).

Participants identified ways in which the QoL tool could be altered to support their ongoing use, such as utilizing prompts to self-monitor if the website had not been accessed in some time or providing a quantification of change since the last completed questionnaire. Interestingly, 2 people took matters into their own hands and adapted the questionnaire to suit their preferences: 1 created a laminated version of the display of the QoL tool results (see Figure 1) on which they marked their daily self-assessment. Another participant created a Microsoft Excel table aggregating their scores from the QoL tool, along with a *diary* column to help pinpoint the cause of changes in QoL. Personalization appeared to support engagement with QoL self-monitoring; in both cases, individuals described the QoL tool as a key component of their ongoing self-management plan:

It’s not just a bunch of scores. This is at the heart of my entire self-management strategy.

Male, aged 35-44 years, BD-I

## Discussion

### Principal Findings

This qualitative study explores the subjective experience of QoL self-monitoring in BD by interviewing individuals who had utilized a BD-specific QoL measure, the QoL tool. This investigation adds to the growing literature on digital monitoring in BD but is the first to comment on a QoL-specific app. Furthermore, although this qualitative study was conducted with a sample of individuals who self-selected into using the QoL tool in the context of a self-management intervention, it has implications for supporting self-reflection and utilizing subjective QoL data in clinical practice.

The breadth of life domains assessed by the QoL tool was identified as an important aspect of engagement with self-monitoring and appears fundamental to other key experiences described herein: assessing a range of domains increases the chances of individuals identifying the areas of strength alongside those in need of remedial action. The perceived value of tracking a range of domains accords with the findings that people with BD are already attempting to log multiple indicators beyond symptoms [7]; however, few apps exist to support this. A systematic review of apps for BD showed that the majority of self-monitoring apps track symptoms, sleep, and medication use [36]. Owing to interest in gauging holistic outcomes and the lack of technologies to support this, the positive responses to a Web-based QoL self-report measure documented in this study validate further study and development of QoL self-assessment tools. Furthermore, given increasing attention to mobile phone–based monitoring of symptoms [5,37,38], complementary QoL-focused apps may be a useful focus of further development.

A key aspect of the experience of QoL self-monitoring was the positive nature of using results to identify the areas of strength. This may be expected given that the QoL tool includes positive rather than negative indicators (ie, individuals are asked about enjoyment and satisfaction, rather than distress and dysfunction). It has been argued that scales, which include positive indicators, enable the detection of flourishing areas, whereas those composed solely of negative indicators tend to underestimate a person’s QoL [39]. Although this does not preclude respondents attending to negative aspects of their circumstances, it may be argued that a broad, positively framed questionnaire is more likely to detect the areas of strength for 2 reasons. First, in accordance with recovery perspectives [40], the broader lens of QoL places de facto emphasis on life domains where people may experience satisfaction and meaning despite the limitations of illness. Second, the QoL tool has positively framed item wording that may promote attention to strengths. Positively framed questions have been used therapeutically to encourage respondents to adopt a shared perspective with the questioner; if a question asks about strengths, individuals will seek evidence of this from their own lives [41]. There are important clinical implications affirming the experience of QoL self-monitoring in this study. First, QoL-focused monitoring may address a noted limitation of symptom tracking, which is the reinforcement of negative symptoms. An analysis of app store reviews of cognitive behavioral therapy apps for depression revealed the primary criticism was the sole focus on negative experiences, with users worrying that this could reinforce negative thinking patterns [42]. Similarly, qualitative studies in BD and psychosis highlights user concerns that attention to symptoms reinforces negative affect [4,25]. Conversely, as suggested by the experiences of participants in this study, tracking *positive* experiences may amplify their emotional and psychological impact or induce a positive reframing of circumstances. This is supported by quantitative research showing an experience sampling intervention focusing on positive affect that reduced depressive symptoms in a sample of patients with major depressive disorder [43,44]. Future quantitative comparisons of the psychological and emotive impact of mood charting versus QoL self-monitoring may help further illuminate their optimal therapeutic apps, such as identifying individuals who may be more likely to be negatively impacted by mood charting or the adjunctive benefits of the more positively oriented QoL monitoring. The second potential clinical app of drawing attention to strengths in broader life domains via reflecting on QoL is fostering the development of a holistic sense of self, a process that is thought to be disrupted in BD [45,46]. Although traditional outcome tracking may also assist with this by helping understand the shifts in mood [47], some individuals may find that it threatens other valued aspects of identity, such as the concept of the *healthy self* [4]. At least one individual in this study expressed a sense that drawing attention to areas of strength through QoL self-monitoring assisted with recovering their identity beyond *someone with bipolar*, which accords with the suggestion that developing balanced and less pessimistic views of the self is a key therapeutic mechanism of psychosocial interventions in BD [2]. Taken together, this suggests possible inherently therapeutic applications of QoL self-monitoring (that is, beyond its possible role in guiding self-management efforts, discussed below), which future studies could explore in more detail.

The potential role of QoL self-monitoring in guiding and encouraging self-management is an important area of future investigation, particularly, as self-management is an increasingly recognized component of treatment in BD [3]. Individuals in this study reported using QoL tool data to identify priority areas for implementing self-directed wellness strategies. This application could be extended to the support of self-management practices and the identification of treatment goals in the clinical context, which has been suggested to improve therapeutic alliance [48] and motivation to engage in treatment [49]. However, this study was generated in the context of self-directed QoL self-monitoring; experiences of sharing data with treating teams were not explored in detail nor were investigations conducted into how health care providers made use of these data. It has been noted that the health care provider’s uncertainty about how to interpret and respond to self-reported data may pose a risk to therapeutic alliance [7] or negatively impact engagement in self-monitoring [25]. Therefore, complementary research on optimal methods for integrating patient-reported QoL data into treatment planning and outcome assessment in the clinical context is needed.

The optimal frequency of self-monitoring in BD is unknown [50]. This study examined self-directed patterns of use, highlighting 2 groups: individuals who engaged with the QoL tool routinely and those who planned to *check in* on an ad hoc basis. Disengagement is common with digital health interventions [22], and although engagement was not formally assessed over an extended period in this study, reported plans for use are promising. Potentially, the lack of a required frequency of QoL monitoring and the strength-focused nature encourage ongoing use, which warrants further investigation. Of note, some participants expressed desires for additional features or themselves made modifications to the QoL tool, suggesting the following: (1) a need for lived experience involvement in the development of future iterations of digital QoL tracking apps (such as user-centered design [51]) and (2) health care providers initiating conversations with users about how self-monitoring can be supported or adapted to meet their unique needs and preferences.

### Limitations

First, the opportunistic sample was drawn from an evaluation of self-management knowledge translation, and as such, they may have had more positive views of QoL self-monitoring and self-management. It is not known whether the experiences described here would generalize to individuals not engaged in psychosocial interventions. In addition, as the overarching study aimed to specifically evaluate the Bipolar Wellness Centre, quantitative data on the frequency of use of the QoL tool were not available to objectively verify self-reported patterns of use. Future research should be conducted to quantify the rates of use of the QoL tool, evaluate the potential for QoL tracking to support engagement in self-management, and investigate ways to support engagement in self-monitoring in a broader sample. Furthermore, purposive sampling of individuals who disengage from psychosocial interventions or QoL self-monitoring may illuminate negative experiences in more detail than this study. Second, the use of the QoL tool was not the primary focus of the interviews, and the data were less rich than the themes explored regarding engagement with the self-management intervention [34] and perceptions of own QoL [33]. We did not probe in depth for prior experiences of mood monitoring or ask participants to contrast this to their experiences of QoL tracking. To address the risk of overinterpretation of the evidence, analysis of the qualitative data was limited to descriptive, literal categories, rather than risk bias by generating interpretative themes [52].

### Conclusions

For the first time, individuals with BD have commented on the experience of engaging with a Web-based tool for QoL self-monitoring. This study highlights the unique characteristics of QoL tracking that were valued by the participants, including the breadth of domains assessed, the ability to affirm strengths, and the link between reflecting on QoL tool results and actions to improve one’s circumstances. A variety of patterns of use were described, with an emphasis on flexibility. It is concluded that QoL self-monitoring shows great potential to support recovery-oriented and self-management interventions and may have an inherently therapeutic quality relevant to the treatment of BD.

## Abbreviations

BD: bipolar disorder
BD-I: bipolar I disorder
BD-II: bipolar II disorder
BD-NOS: bipolar disorder not otherwise specified
CREST.BD: Collaborative Research Team for Psychosocial Issues in Bipolar Disorders
LCM: Life Chart Methodology
QoL: quality of life
